# What is the best electrode setting to elicit motor evoked potentials in the muscles of lower extremities during supratentorial surgery?

**DOI:** 10.1016/j.cnp.2025.10.005

**Published:** 2025-10-26

**Authors:** Fares Komboz, Jan-Bernd Wemhoff, Andrea Szelényi, Beate Kranawetter, Tatiana Chacon, Angelina Nazarenus, Caspar Stephani, Veit Rohde, Tammam Abboud

**Affiliations:** a Department of Neurosurgery, University Medical Center Göttingen, Göttingen, Germany; b Department of Neurosurgery, LMU Klinikum Großhadern, Munich, Germany; c Department of Neurology, University Medical Center Göttingen, Göttingen, Germany; d Department of Anesthesiology, University Medical Center Göttingen, Göttingen, Germany

**Keywords:** Electrode setting, Intraoperative neuromonitoring, Motor evoked potentials, Threshold criterion, Transcranial electrical stimulation

## Abstract

**Objective:**

Eliciting lower extremity transcranial motor evoked potentials (LE-tcMEP) during supratentorial surgery can be challenging, as it often requires high current intensities, bearing the risk of bypass stimulation with false positive results. The aim of this study was to evaluate a new hemispheric electrode combination (C3/4 ↔ Cz + 6), and compare it with established ones, determining the best scalp electrode setting with the minimal motor thresholds (MT) to elicit LE-tcMEP.

**Methods:**

Patients undergoing surgery for supratentorial lesion removal requiring intraoperative neuromonitoring were prospectively included. TcMEP were elicited using montages C1 ↔ C2, C3 ↔ C4, C3/4 ↔ Cz, C3/4 ↔ Cz + 6 and Cz ↔ Cz + 6. MT was established for each muscle and montage to determine the electrode combination with the lowest MT and highest selectivity.

**Results:**

Based on 5880 measurements (70 patients), we found that the mean MT for eliciting LE-tcMEPs was lowest for C3 ↔ C4 (83.0 mA, p = 0.001), followed by C3/4 ↔ Cz + 6 (96.5 mA). Successfully eliciting contralateral LE-tcMEP was best achieved with C3 ↔ C4 (99.2 %, p < 0.0001), followed by C3/4-Cz + 6 (98.9 %). C3/4 ↔ Cz + 6 achieved the highest side selectivity (p < 0.001).

**Conclusion:**

While electrical stimulation at C3 ↔ C4 requires the lowest MT to elicit LE-tcMEPs, stimulation at C3/4 ↔ Cz + 6 was the most selective.

**Significance:**

C3/4 ↔ Cz + 6 may be a superior choice in supratentorial surgery, avoiding bypass-stimulation of deeper (e.g. brainstem) corticospinal fibers.

## 1 Introduction

Transcranial motor evoked potentials (tcMEPs) provide non-invasive, real-time assessment of corticospinal tract (CST) integrity and are therefore a critical component of intraoperative neuromonitoring (IONM) (Kombos et al., 2009, Sala et al., 2007, Szelényi et al., 2005, Taniguchi et al., 1993). In fact, IONM reduces surgery-dependent morbidity as has been extensively demonstrated in brain tumor surgery (Abboud et al., 2018, Guo et al., 2018, Kombos et al., 2001, Motomura et al., 2018, Neuloh et al., 2007, Szelényi et al., 2010, Talacchi et al., 2010). Continuous tcMEP monitoring helps detect potential CST injuries promptly, enabling immediate corrective measures.

Despite its widespread application, eliciting reliable lower extremity tcMEPs (LE-tcMEPs) presents considerable challenges. High current intensities are often required to elicit responses, which can induce unwanted patient movement, complicating the surgical procedure and causing discomfort for the surgeon (Rothwell et al., 1994, MacDonald, 2002, Szelényi et al., 2007). Moreover, excessive stimulation intensity risks activation of deeper white matter fibers, potentially bypassing the surgical site, thereby masking CST injuries (MacDonald et al., 2013, Umemura et al., 2018). Such limitations highlight the need for improved stimulation techniques that provide reliable monitoring with reduced stimulation intensity.

TES montages can be classified into interhemispheric, midline and hemispheric. Interhemispheric stimulation is performed using electrode combinations such as C1 ↔ C2 (median interhemispheric) or C3 ↔ C4 (lateral interhemispheric), which preferentially activate the hand area at lower intensities. Lateral interhemispheric stimulation notably activates ipsi- and contralateral tcMEP alike. Midline stimulation involves electrode combinations like Cz ↔ Cz + 6 or Cz ↔ Oz, reportedly preferentially eliciting LE-tcMEP. In contrast, more focal activation of the hand motor area is achieved through hemispheric stimulation using the electrode combination C3/C4 ↔ Cz.

The current recommendation for electrode placement to elicit “reasonable monitorable responses” in the upper extremities (UE) and LE is C1 ↔ C2, while C3 ↔ C4 or C3/C4-Cz is considered optimal for the distal UE (Deletis, 2002, Szelényi et al., 2007, MacDonald et al., 2013). Cathode placement 6 cm frontal to Cz, labeled Cz + 6, has been shown to be beneficial in LE stimulation (Szelényi et al., 2007). Yet the use of hemispheric stimulation involving a Cz + 6 cathode has not been reported to this date.

The limitations of standard TES protocols for LE-tcMEPs underscore the need for novel stimulation strategies. Hemispheric stimulation with a Cz + 6 cathode may offer advantages in terms of specificity and reduced current intensity, particularly during supratentorial surgeries where excessive stimulation can compromise the safety and accuracy of IONM.

The aim of this study was to evaluate the performance of the novel hemispheric stimulation modality C3/C4-Cz + 6 and compare it to widely used scalp electrode settings. Specifically, we sought to determine whether this approach could lower the minimum current intensity required to elicit reliable LE-tcMEPs during supratentorial surgery.

## 2 Methods

### 2.1 Study design

We conducted a prospective observational study of patients scheduled for supratentorial surgery requiring IONM at our institute between January 2019 and December 2022. Informed consent was obtained from all patients preoperatively. Admission evaluation included magnetic resonance imaging (MRI) and a full neurological examination. Exclusion criteria were preoperative epilepsy, the presence of any motor deficits and a lesion location that would prevent electrode placement (e.g., precentral gyrus). Intraoperative TES was performed to elicit tcMEP during surgery. Postoperative assessment included postoperative imaging (CT and/or MRI) and a full neurological examination at discharge. The study was approved by the local ethics committee of the University Medical Center Göttingen (application number 33/7/19).

### 2.2 Anesthesia

All procedures were performed under general intravenous anesthesia according to local standards (Abboud et al., 2022). The same protocol was followed for all patients, using identical drugs in weight-adjusted doses (Abboud et al., 2017). After induction of anesthesia, all stimulation and recording electrodes were applied to the patient. A bite block (rolled gauze) was placed in the mouth to prevent bite injuries due to contraction of masticatory muscles. Anesthesia was induced and maintained with propofol, analgesia was initiated with sufentanil and continued with remifentanil. Invasive blood pressure and body temperature measurements were taken and monitored during all procedures.

### 2.3 Surgical treatment

Surgery was performed with the primary goal of maximizing tumor resection while preserving brain function. Intraoperative navigation was used in all procedures, and 5-ALA was administered to patients with a probable diagnosis of malignant glioma or metastasis after obtaining informed consent. Depending on the tumor, the goal of the surgery was to remove the 5-ALA enhancing region on fluorescent microscopy, the contrast-enhancing region on neuronavigation for contrast-enhancing tumors and the high-intensity T2 region in non-enhancing tumors. Pathologic diagnosis was based on the 2016 World Health Organization (WHO) classification.

### 2.4 Transcranial electric stimulation

**Fig. 1:**
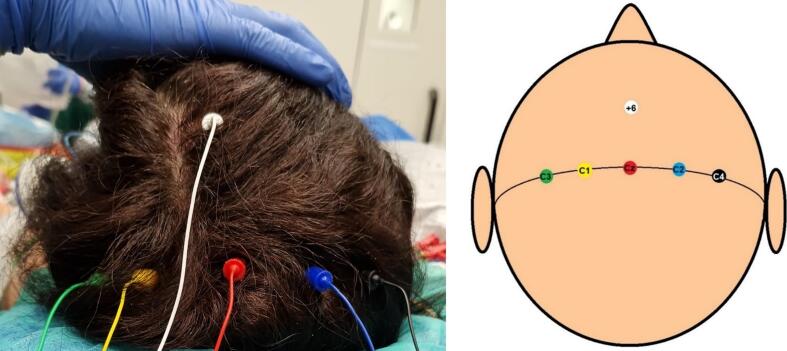
Corkscrew electrode positioning: green: C3, yellow: C1, red: Cz, blue: C2, black: C4, white: Cz + 6. (For interpretation of the references to colour in this figure legend, the reader is referred to the web version of this article.)

MEP monitoring was initiated before head fixation. Corkscrew electrodes (Neurodart, Spes Medica) were placed subcutaneously at C1, C2, C3, C4, Cz and Cz + 6 according to the international 10–20 electroencephalography system (Fig. 1). When separated by a hyphen in the text, the first electrode mentioned in a given pair served as the anode and the second as the cathode, e.g., C1-C2 = C1(anode)–C2(cathode). The double arrow indicates that each electrode in a given pair is used as an anode while the other serves as a cathode in two separate settings. For example, C3 ↔ C4 refers to both C3(anode)-C4(cathode) and C4(anode)–C3(cathode) settings, and C3/4 ↔ Cz + 6 refers to all of C3(anode)-Cz + 6(cathode), C4(anode)-Cz + 6(cathode), Cz + 6(anode)–C3(cathode), and Cz + 6(anode)-C4(cathode) montages. For each patient, stimulation was performed with each of the following combinations: median interhemispheric (C1 ↔ C2), lateral interhemispheric (C3 ↔ C4), hemispheric (C3/4 ↔ Cz and C3/4 ↔ Cz + 6) and midline (Cz ↔ Cz + 6). TES was applied via constant-current stimulation (anodal 5-train stimulation, 0.5 ms individual pulse width, 2 ms interstimulus interval, 220 mA upper intensity limit).

### 2.5 Recording of muscle MEPs

To record tcMEPs, subdermal needle electrodes (Neurodart, Spes Medica) were inserted bilaterally into the abductor pollicis brevis (APB), tibialis anterior (TA) and abductor hallucis (AH) muscles of all patients (100-ms epoch length, 1.5–853 Hz band-pass filter, 10,000x gain). For stimulation and recording, we used ISIS Xpert® and NeuroExplorer (V. 5.1) software (Inomed Medizintechnik GmbH, Emmendingen, Germany). The motor threshold (MT) was defined as the stimulation intensity that elicited tcMEPs with an amplitude ≥ 30 µV from the target muscle within three consecutive trials at a 1-Hz train repetition rate (Abboud et al., 2016). Thresholds levels (in mA) were determined for each connected muscle and for each electrode combination at baseline.

### 2.6 Outcome measures

Stimulation intensities were analyzed to compare the electrode combinations based on the following parameters:

1) The MT (mA) for all recorded muscles in all patients. Since the maximum allowable stimulation intensity was 220 mA, a TES that did not elicit a specific tcMEP was assigned an MT value of 221 mA to account for the presumed increased MT value at this setting.
2) The rate (%) of successful eliciting of contralateral LE-tcMEP per electrode combination.
3) Side selectivity: In each patient and for each hemispheric electrode configuration (the widely used C3/4 ↔ Cz and the new C3/4 ↔ Cz + 6), side selectivity was defined as to whether the contralateral LE muscles (TA and AH) both exhibited lower MT than the ipsilateral hand (APB). The side selectivity of each electrode setting was calculated as the percentage of patients in the total population that exhibited side selectivity for the given electrode setting. For example, if stimulation of patient A with the C3-Cz setting resulted in an MT of 72, 75 and 93 mA, recorded in the right TA, right AH, and left APB, respectively, the setting C3-Cz would be considered side-selective for the right side. The purpose of this outcome parameter was to allow a direct comparison between the two cathodes Cz and Cz + 6.

### 2.7 Statistical analysis

All data were analyzed using SPSS Statistics 27 (IBM SPSS Statistics, Chicago, IL, USA), Microsoft Excel (2013, Microsoft Inc., Seattle, Washington, USA), and SigmaPlot (v12.5, Systat Software Inc., Erkrath, Germany). For both patient-specific variables and MT for each electrode montage, descriptive statistical analysis was performed. Values were presented as mean ± standard deviation (SD). Normal distribution was assessed by the Shapiro-Wilk test. Normally distributed data were presented as mean and standard deviation. Mean MT were compared using a one-way ANOVA with a post-hoc Tukey test.

The second outcome measure was addressed as a binary measure (LE-tcMEP elicited? Yes/no) per muscle per patient. All binary values were listed for each electrode montage, and a Chi-Square test was performed to compare the rate of successful LE-tcMEP between the different electrode settings.

To assess the outcome parameter “side selectivity”, a mixed logistic regression model was applied, where side selectivity was chosen as a binary dependent variable. A random intercepts model was included to control for repeated measures in the same subject.

The significance level was set at a two-tailed p ≤ 0.05. Owing to the exploratory nature of the study, no adjustment for multiple testing was applied.

## 3 Results

### 3.1 Patient demographics

70 patients aged 60 ± 15.8 years were included. Descriptive patient data are listed in Table 1. All received tumor surgery (33 gliomas, 19 meningiomas, 10 metastases, 3 lymphomas, 5 other entities). 31 lesions were left-sided, 35 right-sided, and 4 lesions were bilateral. A total of 5880 measurements were performed.

**Table 1:** Descriptive patient data. 70 patients aged 60 ± 15.8 years were included. 33 (47.1 %) were females, 37 (52.9 %) were males. Mean height was 172.2 ± 9.2 cm, mean weight was 79.6 ± 18.9 kg.

Variable	N	Mean	SD	Min	Max
Age	70	60.07	15.83	20	84
Height (cm)	70	172.2	9.23	158	197
Weight (kg)	70	79.61	18.93	50	140

### 3.2 Mean motor thresholds

Interhemispheric montages: For the median interhemispheric setting C1 ↔ C2, the mean MT was 149 (±44) mA for the ipsilateral LE (TA + AH combined) and 119 (±41) mA for the contralateral LE. The mean ipsi- and contralateral LE-MT was 103 (±34) mA and 83 (±29) mA respectively for the lateral interhemispheric montage C3 ↔ C4.

An independent *t*-test showed no significant difference in mean MT when stimulating the right LE with C1-C2 vs the left LE with C2-C1 (119 vs 111 mA, p = 0.08). However, mean MT were significantly higher for the right LE stimulated with C3-C4 than the left LE stimulated by C4-C3 (102 vs 89 mA respectively, p < 0.001).

Hemispheric montages: The mean LE-MT was 189 (±41) mA (ipsilateral) and 146 (±42) mA (contralateral) for C3/4-Cz, and 186 (±36) mA (ipsilateral) and 97 (±37) mA (contralateral) for C3/4-Cz + 6.

Midline montages: With the midline montage Cz-Cz + 6, a mean MT of 134 (±43) mA was elicited in the left LE and 118 (±37) mA on the right. MT per muscle per electrode setting were detailed in Table 2.

**Table 2:** Mean motor threshold intensity in mA per muscle per given electrode montage. The lowest thresholds recorded for the lower extremities were marked in bold. Abbreviations: TA = tibialis anterior, AH = abductor hallucis, APB = abductor pollicis brevis.

	C1-C2	C2-C1	C3-C4	C4-C3	C3-Cz	C4-Cz	C3-Cz + 6	C4-Cz + 6
TA left	152.49	113.91	102.64	**77.6**	192.44	137.16	190.63	**92.36**
TA right	118.44	141.8	**83.31**	99.3	151.06	186.2	**95.17**	181.44
AH left	151.03	114.31	105.36	**80.96**	187.99	140.91	188.41	**96.21**
AH right	128.2	149.5	**90.16**	106.47	156.81	188	**102.39**	181.83
APB left	131.29	83.1	86.89	46.2	145.41	63.23	163.2	65.76
APB right	78.71	136.41	48.7	89.91	66.74	148.57	65.4	160.04

	**Cz-C3**	**Cz-C4**	**Cz + 6-C3**	**Cz + 6-C4**	**Cz-Cz + 6**	**Cz + 6-Cz**		
TA left	125.94	128.17	198.56	137.73	140.57	173.21		
TA right	132.21	117.21	132.77	193.31	123.86	169.63		
AH left	127.66	132.9	198.94	137.53	141.96	174.59		
AH right	137.37	123.63	138.11	196.19	130.31	173,83		
APB left	143.53	134.56	132.2	108.96	167.5	149,99		
APB right	135.41	131.5	111.67	149.31	156.84	143,36		

A one-way ANOVA performed to compare the mean MT showed a significant difference between different MEP settings (p < 0.0001). A post-hoc analysis showed that, for the contralateral LE, the mean MT was statistically different for each montage comparison: the C3 ↔ C4 exhibited a significantly lower MT (83 mA, p = 0.001), followed by C3/4-Cz + 6 (96 mA, p = 0.001), then C1 ↔ C2 (119 mA, p < 0.0001), Cz-Cz + 6 (134 mA, p = 0.002, both sides combined), and finally C3/4-Cz (146 mA, p = 0.002).

### 3.3 Rate of successful contralateral LE-TcMEP

The total of LE-tcMEP recordings performed in this study was 280 (4 muscles per patient).

Interhemispheric montages: Using C1 ↔ C2, ipsilateral LE-tcMEP could be successfully elicited in 247 muscles (88.2 %) vs 270 (96.4 %) contralaterally. In comparison, the percentual value for C3 ↔ C4 (97.1 % or 272 muscles ipsilaterally and 99.2 % or 278 muscles contralaterally) was higher than for C1 ↔ C2.

Hemispheric montages: For C3/4-Cz, the rate was 58.6 % (164 muscles) on the ipsilateral and 93.2 % (261 muscles) on the contralateral side, whereas it reached respectively 68.6 % (192 muscles) and 98.9 % (277 muscles) on the ipsi- and contralateral LE for C3/4-Cz + 6. Applying the configurations Cz-C3 and Cz-C4 led to a higher rate of successful ipsilateral LE-tcMEP than the contralateral one (Table 3).

**Table 3:** Percentage of patients with a successful eliciting of a given muscle tcMEP by a given electrode setting with a stimulation up to 220 mA (total = 70 patients). Abbreviations: TA = tibialis anterior, AH = abductor hallucis, APB = abductor pollicis brevis.

	C1-C2	C2-C1	C3-C4	C4-C3	C3-Cz	C4-Cz	C3-Cz + 6	C4-Cz + 6
TA left	87.1 %	98.6 %	98.6 %	100.0 %	58.6 %	98.6 %	62.9 %	100.0 %
TA RIght	98.6 %	91.4 %	100.0 %	98.6 %	92.9 %	61.4 %	98.6 %	72.9 %
AH left	87.1 %	95.7 %	95.7 %	98.6 %	55.7 %	94.3 %	65.7 %	98.6 %
AH RIght	92.9 %	87.1 %	98.6 %	95.7 %	87.1 %	58.6 %	98.6 %	72.9 %
APB LEft	91.4 %	98.6 %	100.0 %	100.0 %	87.1 %	100.0 %	85.7 %	98.6 %
APB right	97.1 %	91.4 %	100.0 %	100.0 %	98.6 %	87.1 %	98.6 %	91.4 %

								
	**Cz-C3**	**Cz-C4**	**Cz + 6-C3**	**Cz + 6-C4**	**Cz-Cz + 6**	**Cz + 6-Cz**		
TA left	98.6 %	97.1 %	47.1 %	95.7 %	92.9 %	71.4 %		
TA right	95.7 %	97.1 %	95.7 %	55.7 %	94.3 %	75.7 %		
AH left	94.3 %	95.7 %	47.1 %	94.3 %	91.4 %	62.9 %		
AH right	94.3 %	95.7 %	94.3 %	47.1 %	90.0 %	65.7 %		
APB left	87.1 %	92.9 %	97.1 %	100.0 %	81.4 %	87.1 %		
APB right	95.7 %	91.4 %	98.6 %	92.9 %	87.1 %	87.1 %		

Midline montages: With Cz-Cz + 6, TcMEPs could be elicited in both LE (combined) with a rate of 92.1 % (258 muscles), whereas with Cz + 6-Cz, this occurred for 68.9 % (193 muscles). Detailed success rates per muscle (for a total of 70) per electrode setting are presented in Table 3.

A Chi-Square test was conducted to compare the success rates of contralateral LE stimulation between the different electrode montages. The C3 ↔ C4 setting (278/280) exhibited statistically the highest success rates (p < 0.0001), followed by the C3/4-Cz + 6 montage (277/280, p < 0.0001), and C1 ↔ C2 (270/280, p < 0.0001). C3/4-Cz (261/280, p < 0.0001) as well as Cz-Cz + 6 (258/280, p < 0.0001, both sides combined) showed the lowest success rates.

### 3.4 Side selectivity

The side selectivity parameter was calculated for the interhemispheric (C1 ↔ C2 and C3 ↔ C4) and hemispheric electrode configurations (C3/4 ↔ Cz and C3/4 ↔ Cz + 6) as previously defined (s. Methods – outcome measures). It was highest for C3/4 ↔ Cz + 6, reaching 127/140 (90.7 %) for both sides, followed by C3 ↔ C4 (81/140, 57.9 %), C1 ↔ C2 (79/140, 56.4 %) and finally C3/4 ↔ Cz (62/140, 44 %). To compare both hemispheric montages, a mixed logistic regression model was applied, with side selectivity as a binary dependent variable. The cathode Cz + 6 was associated with a significantly higher side selectivity (p < 0.001), implying that the C3/4 ↔ Cz + 6 setting allows a stimulation of the contralateral LE with a higher side selectivity than the standard C3/4 ↔ Cz setting (Table 4).

**Table 4:** The side specificity of each montage was defined as to whether the contralateral lower extremity muscles (TA and AH) both exhibited lower MT as the ipsilateral hand (APB). The rate in the total patient population (n = 70) was then calculated. The C3/4 ↔ Cz + 6 setting showed the highest rate of side specificity.

Electrode setting	Side specificity rate (/70)
C1-C2	35
C2-C1	44
C3-C4	36
C4-C3	45
C3-Cz	26
C4-Cz	36
C3-Cz + 6	63
C4-Cz + 6	64

## 4 Discussion

The results of this study demonstrate distinct differences in the performance of various corkscrew electrode configurations for eliciting LE-tcMEPs. Specifically, the analysis focused on stimulation intensity, successful stimulation rate, and side selectivity.

The lowest stimulation intensity required to elicit LE-tcMEPs was achieved using C3 ↔ C4 (mean MT = 83 mA, p = 0.001), followed by C3/4 ↔ Cz + 6 (at 97 mA). This indicates that the C3 ↔ C4 configuration provides the most efficient stimulation in terms of the minimum current required to achieve reliable responses. The rate of successful LE-tcMEP was highest when using C3 ↔ C4 (278/280 attempts, p < 0.0001) and C3/4 ↔ Cz + 6 (277/280), indicating that both montages are highly effective for generating reliable tcMEPs. C3/4 ↔ Cz + 6 demonstrated superior side selectivity (90.7 % bilaterally) compared to 57.9 % for C3 ↔ C4. When evaluating both hemispheric montages (C3/4 ↔ Cz + 6 and C3/4 ↔ Cz), statistical analysis revealed that Cz + 6 cathode placement was associated with significantly higher side selectivity (p < 0.001) compared to Cz.

**Fig. 2:**
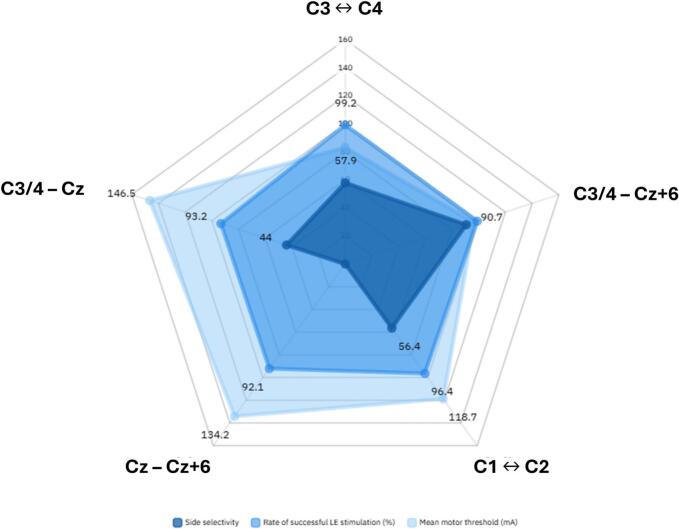
Graphic representation of the performance of each electrode montage. The summarized results highlight differences in stimulation intensity, successful stimulation rate, and side selectivity between the electrode configurations. In light blue: the mean motor threshold in mA; in blue: the rate of successful eliciting of contralateral lower extremity tcMEP with stimulation intensity up to 220 mA (%); in dark blue: the side selectivity (%). To note, the electrode combination placement on this figure is random and is not related to the actual electrode position on the scalp. (For interpretation of the references to colour in this figure legend, the reader is referred to the web version of this article.)

Overall, these findings suggest that while C3 ↔ C4 provides the most efficient stimulation with the lowest intensity and highest success rate, C3/4 ↔ Cz + 6 offers superior side selectivity. Cz + 6 cathode placement could enhance the precision of motor pathway monitoring, which may be particularly valuable during supratentorial surgery (Fig. 2).

### 4.1 Interhemispheric vs hemispheric stimulation

A recent study analyzing tcMEP variations during supratentorial glioma surgery showed a correlation between decreasing tcMEP intensity and transient/permanent neurological deficits (Kuwano et al., 2024). Here, only C3 ↔ C4 stimulation (constant voltage stimulation in the range of 400–600 V) was performed. Of 6 patients with a permanent postoperative deficit, one maintained his MEP amplitude throughout the surgery, and of 15 patients with a transient postoperative deficit with moderate improvement, 3 had a decrease in MEP amplitude of less than 50 % (Kuwano et al., 2024). These results underscore the limitations of C3 ↔ C4 stimulation during supratentorial tumor surgery, as this setting carries the risk of false negative results due to the deep white matter activation, underpassing current propagation beyond the damaged area and consequent masking of the motor fiber damage (MacDonald et al., 2013, Szelényi et al., 2003, Umemura et al., 2018).

The C1 ↔ C2 montage, a quite popular and reasonable electrode placement in neurosurgery, allows concentrated targeting of both hand and leg areas of the motor cortex, while inducing less contraction of the jaw and neck musculature than C3 ↔ C4 (Cohen and Hallett, 1988, MacDonald, 2002, Szelényi et al., 2007). However, the electrode proximity results in an electrical shunt, i.e. a greater distribution of the electrical current across the scalp, limiting the effective brain-activating current (Holdefer et al., 2006). Conversely, hemispheric stimulation, most performed as C3/C4-Cz TES, allows highly selective, unilateral stimulation of the facial and brachial muscles. However, eliciting LE-tcMEP with this setting is more challenging (Szelényi et al., 2007). In this study, we investigated the potential of hemispheric TES using a Cz + 6 cathode in an attempt to combine hemispheric and midline TES. This allowed us to refine the selectivity of hemispheric stimulation as well as the LE coverage facilitated by the cathode placement.

### 4.2 Novel electrode settings

In a recent study, Yamada et al. showed that C1-C4/C2-C3 stimulation allowed a side selective unilateral stimulation of the motor cortex in 97 % of cases (Yamada et al., 2023). This setup solves the shunting problem of the C1 ↔ C2 stimulation while enhancing the advantages of the C3 ↔ C4 electrodes, all while remaining highly side selective. However, a direct comparison with conventional techniques and a clear clinical utility of this setting have yet to be established (Yamada et al., 2023).

An additional observation from our team was the tendency of inverted hemispheric stimulation Cz-C3/C4 (anodal Cz) to elicit lower MT for the extremities ipsilateral to the cathode (table 2). A description of such montages and their efficiency has, to our knowledge, not been described in previous literature. Further follow-up studies including the use of finite element modeling (FEM) have yet to further clarify such novel observations.

### 4.3 Lower extremity monitoring

LE-tcMEP can be challenging to elicit because standard electrode montages often require higher current intensities. This, in turn, results in greater intraoperative patient contractions, potentially disturbing the surgeon during microsurgery.

Using FEM, Tomio et al. were able to illustrate the propagation of the electric fields in the brain for different tcMEP settings. They showed that compared to C1 ↔ C2 and C3 ↔ C4, the Cz ↔ inion montage more effectively covered the pyramidal tract fibers of the LE (Tomio et al., 2016). This is additionally supported by evidence that pyramidal tract fibers exhibit systematic differences in their stimulation thresholds (Stephani and Koubeissi, 2015). Another combined FEM and clinical study showed a superiority of linked quadripolar (C1-M3-C2-M4) montages to C1-C2 and M3-M4 montages regarding UE and LE monitoring (Guo et al, 2023). Szelényi et al. recorded the most focal stimulating electrode montages for the contralateral TA with the setting Cz-Cz + 6 (Szelényi et al., 2007). Other study groups rely on DCS for lower extremity MEP monitoring during vascular neurosurgery of the anterior circulation (Maruta et al., 2012).

In this study, we observed a low stimulation intensity to elicit LE-tcMEPs for the setting C3/4 ↔ Cz + 6, combined with a high side selectivity for the contralateral TA and AH. For these reasons, we believe that C3/4 ↔ Cz + 6 may be superior in supratentorial surgery to elicit UE- and LE-tcMEP without inducing strong intraoperative contractions while avoiding false negative observations due to deeper white matter stimulation underpassing cortical lesions.

### 4.4 Limitations and perspectives

This study proposes a new stimulation paradigm for TES during supratentorial surgery. Correlation of intraoperative recordings with postoperative neurological outcome has yet to be determined. Likewise, it would be interesting to display the propagation of electric fields generated by C3/4 ↔ Cz + 6 using FEM. These experiments were beyond the scope of this study and should be addressed in future investigations.

The observed difference between MT on the left and right side using the C3 ↔ C4 montage could not be explained by the patient selection (comparable number of lesions on the left and the right side, no motor deficits). However, the observed difference was deemed not critical to the interpretations of the pooled results highlighted in this study.

## 5 Conclusion

Establishing TES settings that provide efficient, safe and reliable LE-tcMEP recording can be tricky but remains essential in supratentorial surgery. In our study, the C3/4 ↔ Cz + 6 setting achieved the highest stimulation selectivity with low stimulation intensities, while preserving a high safety profile with a very low risk of subcortical lesion bypass.

## CRediT authorship contribution statement

**Fares Komboz:** Data curation, Formal analysis, Writing – original draft. **Jan-Bernd Wemhoff:** Data curation, Formal analysis. **Andrea Szelényi:** Validation. **Beate Kranawetter:** Data curation. **Tatiana Chacon:** Data curation. **Angelina Nazarenus:** Data curation. **Caspar Stephani:** Project administration. **Veit Rohde:** Validation. **Tammam Abboud:** Conceptualization, Project administration, Supervision.
